# Epidemiological trends and spatiotemporal distribution of syphilis in Nagasaki Prefecture, Japan, 2014–2023

**DOI:** 10.3389/fpubh.2026.1720605

**Published:** 2026-05-12

**Authors:** Binghua Tu, Guoxi Cai, Yixiao Lu, Quwen Li, Kiyoshi Aoyagi, Kazuhiko Arima, Satoshi Mizukami, Hiromu Ito, Hiroaki Arima, Maiko Hasegawa, Rieko Nakao, Masaya M. Saito, Shouhei Takeuchi, Kouichi Morita, Jianfeng Xie, Fei He

**Affiliations:** 1 Department of Epidemiology and Health Statistics, School of Public Health, Fujian Medical University, Fuzhou, Fujian, China; 2 Department of Public Health and Hygiene Research, Nagasaki Prefectural Institute of Environment and Public Health, Nagasaki, Japan; 3 Department of Public Health, Nagasaki University Graduate School of Biomedical Sciences, Nagasaki, Japan; 4 Department of International Health and Medical Anthropology, Institute of Tropical Medicine (NEKKEN), Nagasaki University, Nagasaki, Japan; 5 Yunnan Center for Disease Control and Prevention, Yunnan Academy of Preventive Medicine, Kunming, Yunnan, China; 6 Fujian Provincial Center for Disease Control and Prevention, Fuzhou, Fujian, China; 7 Fujian Provincial Key Laboratory of Zoonosis Research, Fuzhou, Fujian, China; 8 Fujian Provincial Academy of Preventive Medicine, Fuzhou, Fujian, China; 9 Community Health Promotion Division, Health &; Welfare Department, Nagasaki Prefectural Government, Nagasaki, Japan; 10 Department of Public Health Nursing, Graduate School of Biomedical Sciences, Nagasaki University, Nagasaki, Japan; 11 Department of Information Security, Faculty of Information Systems, Nagasaki University, Nagasaki, Japan; 12 Department of Nutrition Science, Faculty of Nursing and Nutrition, Nagasaki University, Nagasaki, Japan; 13 Department of Virology, Institute of Tropical Medicine (NEKKEN), Nagasaki University, Nagasaki, Japan; 14 Dejima Infectious Disease Research Alliance, Nagasaki University, Nagasaki, Japan

**Keywords:** COVID - 19, epidemiological trend, incidence, Japan, Syphilis

## Abstract

**Introduction:**

Syphilis incidence has increased sharply in high-income countries since 2012, including Japan. Nagasaki Prefecture has not previously been subject to detailed epidemiological analysis of syphilis. This study aimed to characterize the epidemiological trends, demographic characteristics, and spatiotemporal distribution of syphilis in Nagasaki Prefecture from 2014 to 2023.

**Methods:**

A descriptive epidemiological study was conducted using 10-year infectious disease surveillance data from Nagasaki Prefecture. Cases were analyzed by time, person, and place distribution. Joinpoint regression analysis was performed to identify significant changes in incidence trends and calculate the annual percentage change (APC). Compound annual growth rates (CAGR) were calculated for overall and stage-specific incidence.

**Results:**

A total of 423 syphilis cases were reported, with the overall incidence rate increasing from 1.26 per 100,000 in 2014 to 11.81 per 100,000 in 2023, representing a 9.4-fold increase with a CAGR of 28.1%. Joinpoint regression identified 2021 as a significant inflection point, with the APC accelerating from 11.83% during 2014–2021 to 99.69% during 2021–2023. Young adults aged 18-34 years showed the most pronounced increase (from seven cases in 2014 to 75 cases in 2023, a 10.7-fold increase). Among cases with documented exposure history, 42.1% (82/195) reported sex industry involvement. Cases were geographically concentrated in Nagasaki City, Sasebo, Isahaya, and Omura.

**Conclusion:**

These findings highlight the disproportionate burden among young adults and the substantial role of sex industry exposure. Targeted screening, integrated HIV-syphilis testing, and strengthened urban surveillance should be prioritized.

## Introduction

Syphilis, one of the most significant sexually transmitted infections (STIs) globally, is caused by the spirochete *Treponema pallidum* (TP), with infected individuals serving as the primary source of transmission (1). The disease is highly contagious; early manifestations include chancres and characteristic syphilitic rashes (2). Although syphilis can be effectively treated with penicillin, if left untreated, it progresses through distinct clinical stages. In Japan's surveillance system, cases are classified as: primary syphilis (characterized by chancres), secondary syphilis (systemic manifestations including characteristic rashes), asymptomatic syphilis (latent infection without clinical symptoms), and late syphilis (tertiary manifestations or confirmed late-stage infection). Primary and secondary syphilis represent the most infectious stages and occur within the 1 year of infection, making them critical indicators of recent transmission for public health surveillance (3). Acute neurosyphilis may also develop during this period. In later stages, syphilis can lead to severe complications, including neurological, cardiovascular, or musculoskeletal diseases (4). In Japan, syphilis is regarded as a serious public health concern under the Infectious Disease Control Law, which was initially enacted in 1999 and updated in 2007 (5). Since 2012, syphilis incidence rates have sharply increased in high-income countries, including Japan (6). In recent years, the COVID-19 pandemic has led to significant behavioral changes, which have also affected the trends in sexually transmitted infections (7). Nagasaki Prefecture, located at the western tip of Japan and in close geographic proximity to the Asian continent, has not previously been the subject of detailed epidemiological analysis of syphilis. Using ten-year infectious disease surveillance data from Nagasaki Prefecture (2014–2023), this study characterized the epidemiological trends and spatiotemporal distribution of syphilis, with particular attention to changes occurring before and after the COVID-19 pandemic.

## Methods

**Study setting:** Nagasaki Prefecture is in the northwest part of the Kyushu area and consists of four peninsulas centered around Omura Bay (Kitamatsuura, Nishisonogi, Nagasaki, Shimabara) and three remote islands (Goto, Iki, and Tsushima). The total population of Nagasaki Prefecture declined from approximately 1,400,000 in 2014 to 1,266,334 in 2023; approximately 34% of residents were aged 65 years or older as of 2023.

**Data sources:** This was a 10-year retrospective observational study covering data from Nagasaki Prefecture between 2014 and 2023. We utilized surveillance data provided by the Nagasaki Prefectural Institute of Environment and Public Health. In Japan, syphilis was classified as a notifiable disease under Category V Infectious Diseases, in accordance with the Act on the Prevention of Infectious Diseases and Medical Care for Patients with Infectious Diseases, which was enacted in 1999 and updated in 2007. According to the law, syphilis cases were required to be reported to local public health centers within 1 week of diagnosis (5). The reportable case definition required a positive result from both a specific treponemal test (e.g., *T. pallidum* hemagglutination assay or latex agglutination) and a non-specific treponemal test (e.g., rapid plasma reagin or latex agglutination), or laboratory identification of T. pallidum (e.g., India ink or Giemsa staining, and/or PCR detection of the bacterial genome from skin lesions). Physicians were legally required to report all such cases to the local public health center, which then forwarded the data to the national level through the National Epidemiologic Surveillance of Infectious Diseases system. For this study, de-identified surveillance data were provided by the Nagasaki Prefectural Institute of Environment and Public Health for research purposes.

**Case definition and inclusion criteria:** Cases were defined according to Japan's notifiable disease criteria under Category V Infectious Diseases, requiring either: (1) positive results from both a specific treponemal test (e.g., TPHA, TP-PA, FTA-ABS) and a non-specific treponemal test (e.g., RPR, VDRL); or (2) laboratory identification of T. pallidum from clinical specimens (e.g., India ink or Giemsa staining, and/or PCR detection). All syphilis cases reported in Nagasaki Prefecture between January 1, 2014, and December 31, 2023, were included in this study without exclusion. Cases were classified into four categories based on clinical staging: primary syphilis, secondary syphilis, asymptomatic (latent) syphilis, and late (tertiary) syphilis.

In Japan's NESID system, syphilis cases are classified into four categories based on clinical presentation and timing:

(1) Primary syphilis: characterized by chancres at the infection site, typically occurring within 3 weeks of infection.

(2) Secondary syphilis: systemic manifestations including skin rashes and mucocutaneous lesions, typically occurring 4–10 weeks after infection.

(3) Asymptomatic (latent) syphilis: serologically confirmed infection without clinical manifestations, representing latent infection at any stage.

(4) Late syphilis: includes late latent syphilis and tertiary manifestations (cardiovascular, neurological, or gummatous disease), typically occurring years after initial infection.

**Statistical Analysis:** The characteristics of reported syphilis cases were summarized using median and interquartile range (IQR) for continuous variables, and frequency with proportions for categorical variables. Data were stratified by sex, and statistical significance was evaluated using the Chi-square test for categorical variables or the Mann–Whitney *U* test for continuous variables. A significance level of *P* < 0.05 was applied. The annual notification rate of reported syphilis cases (for all four notification categories) and the sex-specific annual notification rates across different age groups were calculated as the number of reported cases per 100,000 inhabitants. Joinpoint regression analysis was conducted to examine trends in syphilis incidence and calculate the annual percentage change (APC). All statistical analyses were performed using SPSS software (version 26.0), while trend analyses were conducted using the Joinpoint Regression Program (version 4.8.0.1, April 2020; Statistical Research and Applications Branch, National Cancer Institute, USA) (8). Crude incidence rates were calculated using mid-year population estimates obtained from Nagasaki Prefecture's official population statistics as denominators. Age-specific and sex-specific rates were calculated using corresponding population subgroups. Age standardization was not performed as the primary objective was to describe the actual disease burden in the population. Geographic distribution was assessed by aggregating reported cases by administrative district (city/town level) and calculating cumulative case counts for each area.

**Calculation of growth rates:** The compound annual growth rate (CAGR) was calculated using the formula: CAGR = (End Value / Start Value)^∧^(1/n) - 1, where n represents the number of annual intervals between the first and last year of observation (*n* = 9 for the period 2014–2023). Joinpoint regression analysis was performed to identify statistically significant changes in incidence trends, with the optimal number of joinpoints determined using permutation testing (*P* < 0.05). The annual percentage change (APC) with 95% confidence intervals was calculated for each identified trend segment.

**Clinical trial registration:** not applicable.

**Ethics:** This study was approved by the Research Ethics Committee of the Nagasaki Prefectural Institute of Environment and Public Health (No. 2023-13-1). The study involved secondary analysis of de-identified surveillance data, and no direct contact with human subjects was undertaken. The requirement for individual informed consent was waived by the ethics committee. The study was conducted in accordance with the principles of the Declaration of Helsinki.

## Results

**Figure 1:**
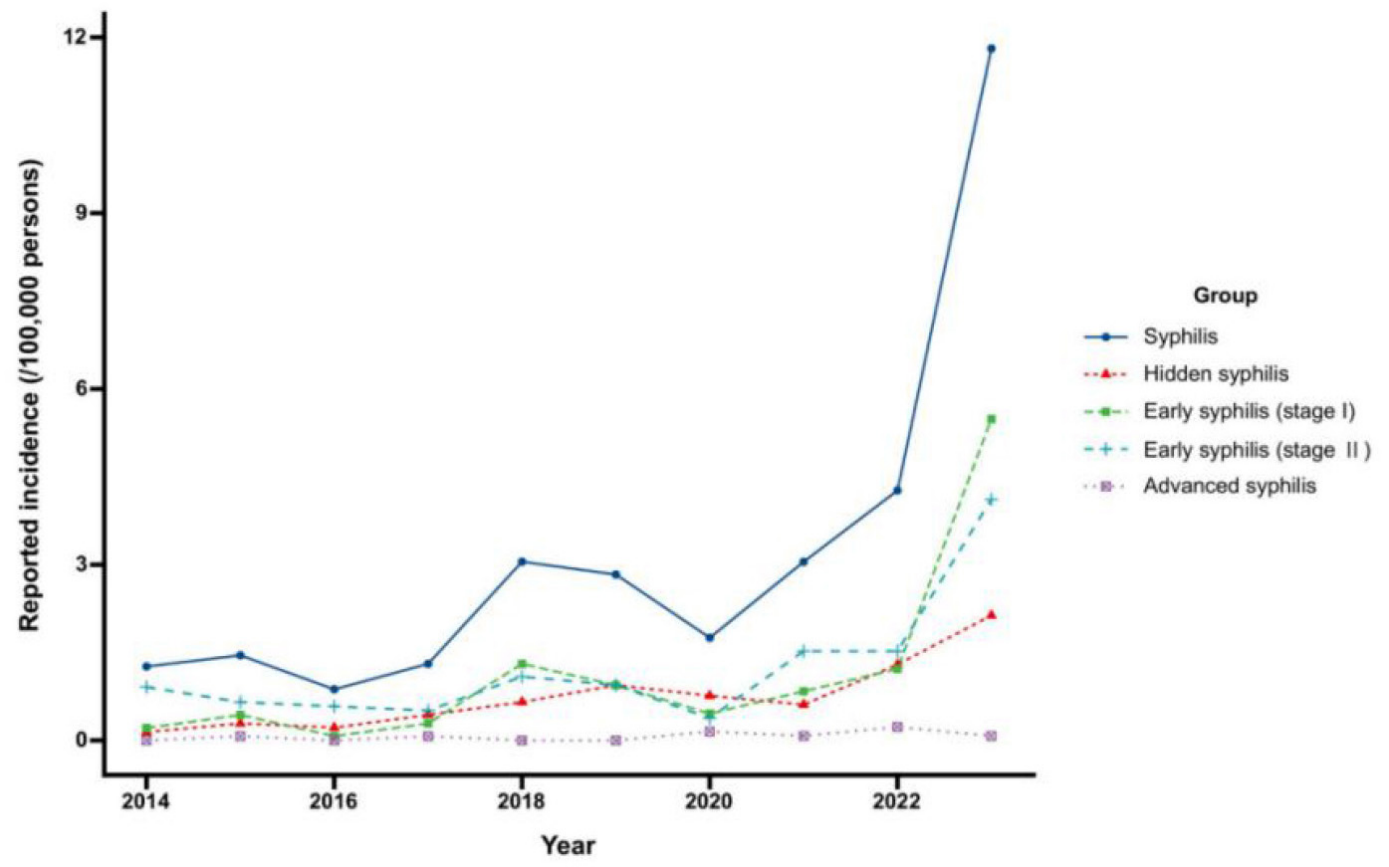
The reported incidence of syphilis from 2014 to 2023.

**Figure 2:**
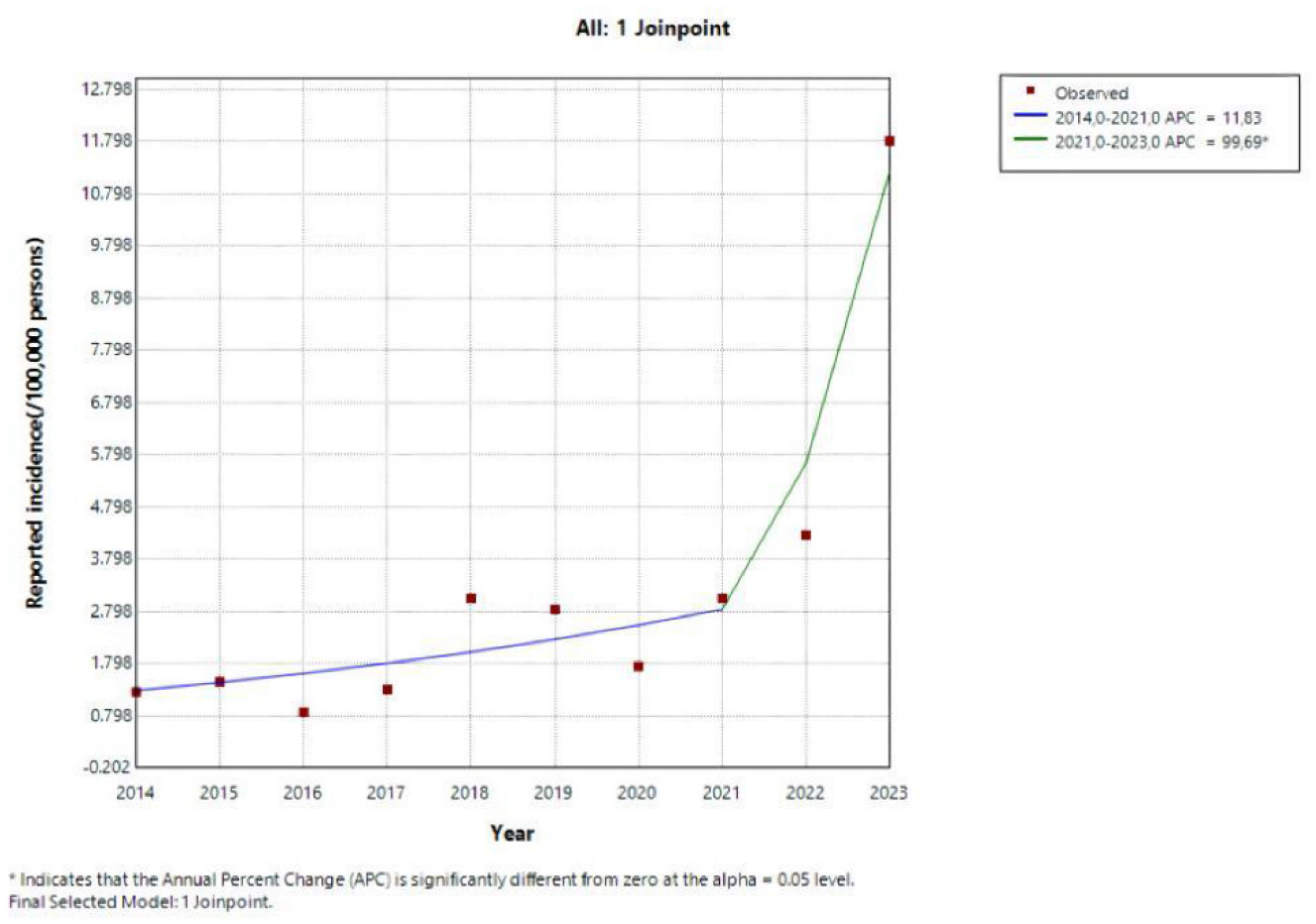
Trends of syphilis reported incidence in Nagasaki from 2014 to 2023.

**Epidemic Trends:** Over the 10-year study period, 423 syphilis cases were reported in Nagasaki Prefecture, of which 289 (68.3%) occurred in males (Figure 1). The overall notification rate increased from 1.26 per 100,000 in 2014 to 11.81 per 100,000 in 2023, representing a 9.4-fold increase with a CAGR of 28.1% (*P* < 0.001). The annual case count rose from 18 in 2014 to 155 in 2023 (8.6-fold); cases in the 18–34 age group increased from 7 to 75 (Figure 2). Growth rates varied substantially by disease stage: primary syphilis (CAGR 43.9%, from 0.21 to 5.49 per 100,000), asymptomatic syphilis (35.3%, from 0.14 to 2.13 per 100,000), and secondary syphilis (18.2%, from 0.91 to 4.11 per 100,000). Late syphilis increased from 0 in 2014 to 0.076 per 100,000 in 2023. Joinpoint regression identified 2021 as a statistically significant inflection point (*P* < 0.05), dividing the observation period into two distinct phases with markedly different growth trajectories: the APC was 11.83% during 2014–2021 and accelerated markedly to 99.69% during 2021–2023 (Figure 2). To examine changes associated with this inflection, we compared characteristics before (2014–2020, 7 years, *n* = 172 cases) and after (2021–2023, 3 years, *n* = 251 cases) this breakpoint. Average annual case numbers increased 3.4-fold, from 24.6 to 83.7 cases per year. The median age decreased from 38 years (IQR: 29–48) to 33 years (IQR: 25–47), with the proportion of cases aged 18–34 years increasing from 39.5% (68/172) to 51.8% (130/251) (*P* = 0.017). The combined proportion of primary and secondary syphilis showed an increasing trend from 70.3% (121/172) to 76.9% (193/251) *(P* = 0.162).

**Figure 3:**
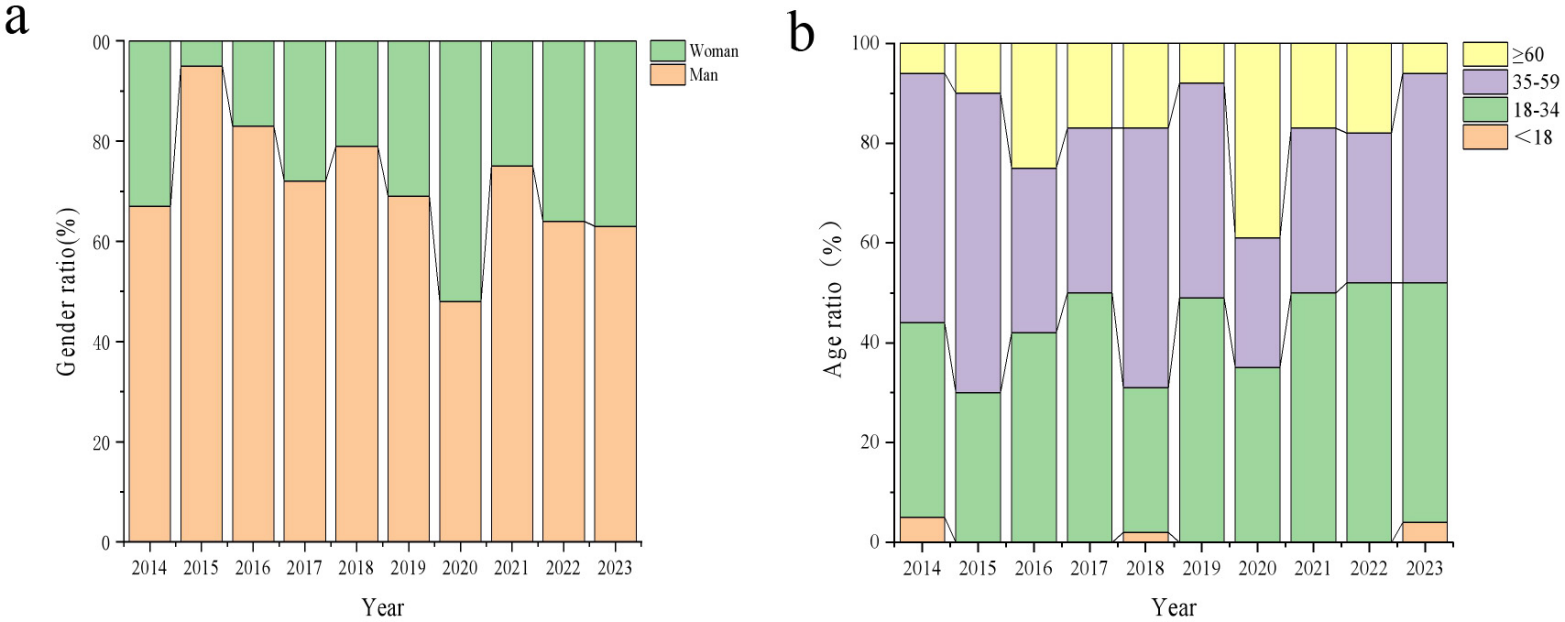
The gender and age distribution of syphilis from 2014 to 2023. **(a)** Changes in gender composition; **(b)** Changes in age composition.

**Population Distribution:** From 2014 to 2023, the male-to-female ratio of syphilis cases decreased from 2:1 to 1.72:1 (Figure 3a). The proportion of individuals under 18 years old decreased from 5.56 to 3.87%, while the proportion of individuals aged 18 to 34 years increased from 38.89 to 48.39%. In contrast, the proportion of individuals aged 35 to 59 years decreased from 50.0 to 41.94%. Additionally, the proportion of individuals aged 60 years and older increased slightly from 5.56 to 5.81% (Figure 3a). A statistically significant difference was observed in the age distribution of reported syphilis cases between men and women from 2014 to 2023 (*P* < 0.001). Notably, the number of syphilis cases among men in the 35–59 age group was 139, markedly higher than the 32 cases reported in women. Furthermore, the number of syphilis cases in men aged 60 years and older was 1.57 times higher than in women (Figure 3b).

**Figure 4:**
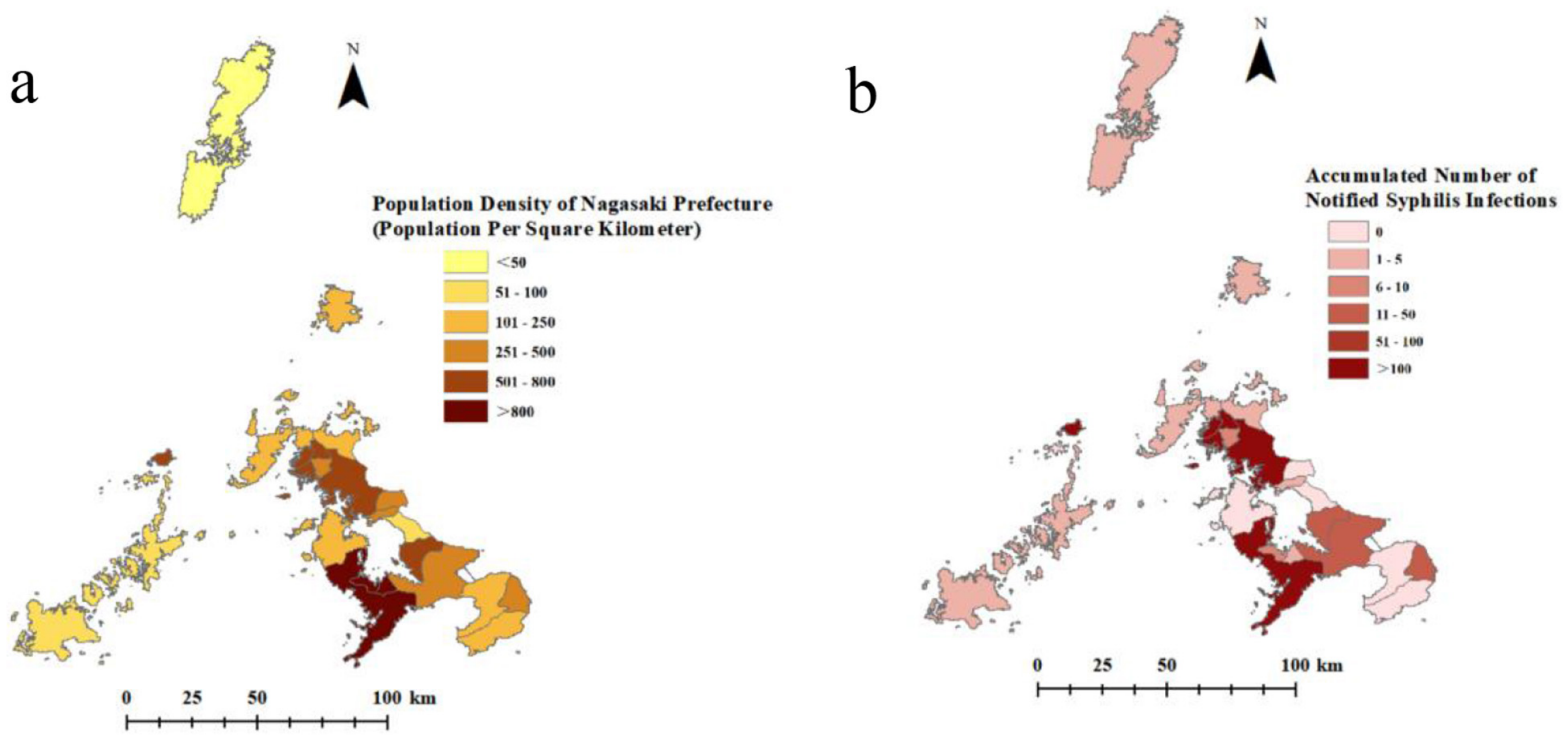
Population distribution and accumulated syphilis infections from 2014 to 2023 in Nagasaki Prefecture. **(a)** Population density per square kilometer in Nagasaki Prefecture, Japan, in 2020; **(b)** Cumulative reported cases of syphilis infection by region, 2014–2023.

**Regional Distribution:** From 2014 to 2023, a total of 423 syphilis infections were reported across 21 districts (13 cities and 8 machis) in Nagasaki Prefecture. The population is concentrated in urban areas, particularly in the cities of Nagasaki, Sasebo, Omura, and Isahaya, which have high population densities (Figure 4a). The distribution of reported syphilis infections in Nagasaki Prefecture was uneven (Figure 4b); while most cases were reported from densely populated areas, it is noteworthy that Tsushima City, despite its very low population density (38 people/km^2^), reported 4 cases of syphilis infections.

**Subgroup Analysis by Age and Risk Factors:** Regarding sex industry involvement, data on exposure history were systematically collected starting from 2019. Among the 195 cases with documented exposure history (excluding those with unknown status), 82 cases (42.1%) reported a history of sex industry involvement. Male cases were significantly more likely to report using commercial sex services (81/135, 60.0%) compared to female cases (1/60, 1.7%; *P* < 0.001). Additionally, 10 cases (2.4%) reported being sex workers, with a higher proportion among females (8/134, 6.0%) than males (2/289, 0.7%; *P* < 0.01).

As shown in Table 1, significant differences were observed between male and female cases in age distribution, disease type, sex industry history, and infection area (all *P* < 0.05). Notably, asymptomatic syphilis was more prevalent among females (39.6% vs. 16.3%), while primary syphilis predominated among males (42.9% vs. 19.4%).

**Table 1:** Characteristics of 423 reported syphilis infections in Nagasaki Prefecture, 2014 to 2023, stratified by sex.

Characteristics	Overall	Male	Female	χ^2^ test/ Mann–Whitney *U* test
	*N* = 423	*N* = 289	*N* = 134	*P*-value
**Age, median (IQR)**	36 (26–67.5)	38 (28–49)	29 (23–45)	<0.001
Age group, *n* (%)	
<18	8 (1.9)	2 (0.7)	6 (4.5)	<0.001
18–34	190 (44.9)	115 (39.8)	75 (56.0)	
35–59	171 (40.4)	139 (48.1)	32 (23.9)	
≥60	54 (12.8)	33 (11.4)	21 (15.6)	
Disease type, *n* (%)	
Asymptomatic syphilis	100 (23.6)	47 (16.3)	53 (39.6)	<0.001
Primary syphilis	150 (35.5)	124 (42.9)	26 (19.4)	
Secondary syphilis	164 (38.8)	112 (38.8)	52 (38.8)	
Late syphilis	9 (2.1)	6 (2.0)	3 (2.2)	
HIV co-infection, *n* (%)	
Yes	6 (1.4)	5 (1.7)	1 (0.8)	<0.01
No	44 (10.4)	21 (7.3)	23 (17.1)	
Unknown	373 (88.2)	263 (91.0)	110 (82.1)	
Pregnancy, *n* (%)	
Yes	6 (1.4)	0 (0)	6 (4.5)	<0.001
No	417 (98.6)	289 (100.0)	128 (95.5)	
Propagation mode, *n* (%)	
Sexual	130 (30.7)	99 (34.3)	31 (23.1)	<0.05
Blood-borne	0 (0)	0 (0)	0 (0)	
Maternal-neonatal	0 (0)	0 (0)	0 (0)	
Unknown	293 (69.2)	190 (65.7)	103 (76.9)	
Sex worker experience^*^, *n* (%)	
Yes	10 (2.4)	2 (0.7)	8 (6.0)	<0.01
No	188 (44.4)	136 (47.1)	52 (38.8)	
Unknown	225 (53.2)	151 (52.2)	74 (55.2)	
Sex industry history^†^, *n* (%)	
Yes	82(19.4)	81 (28.0)	1 (0.8)	<0.001
No	113 (26.7)	54 (18.7)	59 (44.0)	
Unknown	228 (53.9)	154 (53.3)	74 (55.2)	
Infection area, *n* (%)	
Nagasaki prefecture	219 (51.8)	125 (43.3)	94 (70.2)	<0.001
Outside Nagasaki prefecture	103 (24.3)	92 (31.8)	11 (8.2)	
Unknown	101 (23.9)	72 (24.9)	29 (21.6)	

^*^Includes both direct sex workers and adult entertainment industry workers. ^†^Includes clients of direct sex services and patrons of adult entertainment venues.

Table 2 summarizes the main clinical manifestations of reported syphilis cases. Roseola and chancres were the most frequently reported findings. Clinical manifestations were more common in males and in patients aged 18–59 years, whereas multiple concurrent complications were mainly observed in late syphilis.

**Table 2:** Clinical symptoms of 423 reported syphilis infections by sex, age group, and site of disease, Nagasaki Prefecture, 2014–2023.

Characteristics	Primary chancre	Hard chancre	Inguinal lymphadenopathy	Roseola	Papular syphilis rash	Flat warts	Other
Sex, *n* (%)	
Male	84 (84.0)	90 (86.5)	59 (80.8)	78 (69.0)	35 (71.4)	7 (50.0)	10 (71.4)
Female	16 (16.0)	14 (13.5)	14 (19.2)	35 (31.0)	14 (28.6)	7 (50.0)	4 (28.6)
Age, median (IQR)	31 (25–44)	36.5 (26–47.25)	35 (25–44)	36 (26–46)	38 (27–45)	33.5 (22.5–48.5)	52.5 (44.5–76.25)
Age group, *n* (%)	
<18	3 (3.0)	1 (1)	4(5.5)	2 (1.8)	1 (2.1)	0 (0.0)	0 (0.0)
18–34	52 (52.0)	45 (43.3)	31 (42.5)	51 (45.1)	18 (36.7)	7 (50.0)	3 (21.4)
35–59	40 (40.0)	51 (49.0)	34 (46.5)	52 (46.1)	23 (46.9)	5 (35.7)	5 (35.7)
≥60	5 (5.0)	7 (6.7)	4 (5.5)	8 (7.0)	7 (14.3)	2 (14.3)	6 (42.9)
Site of disease, *n* (%)	
Genitals	75 (88.2)	77 (89.5)	–	–	–	–	–
Anus	1 (1.2)	2 (2.3)	–	–	–	–	–
Lips	3 (3.5)	1 (1.2)	–	–	–	–	–
Oropharynx	4 (4.7)	4 (4.6)	–	–	–	–	–
Others	2 (2.4)	2 (2.4)	–	–	–	–	–

Table 3 presents characteristics by disease stage. Male predominance was most pronounced for primary syphilis (82.7%) and secondary syphilis (68.3%), whereas asymptomatic syphilis was more common among females (53.0%). The median age differed by disease stage: asymptomatic syphilis (36 years), primary syphilis (33 years), secondary syphilis (37 years), and late syphilis (53 years). Primary and secondary syphilis, which are key indicators of recent transmission, were predominantly observed in younger patients, particularly those aged 18–34 and 35–59 years. Across disease stages, the RPR card test and TP-PA were the most frequently used diagnostic methods.

**Table 3:** Characteristics of 423 reported syphilis infections in Nagasaki Prefecture, 2014–2023, stratified by disease type.

Characteristics	Asymptomatic syphilis	Primary syphilis	Secondary syphilis	Late syphilis
Sex, *n* (%)	
Male	47 (47.0)	124 (82.7)	112 (68.3)	6 (66.7)
Female	53 (53.0)	26 (17.3)	52 (31.7)	3 (33.3)
**Age, median (IQR)**	36 (27–61)	33 (25–45)	37 (26–46)	53 (44–80)
Age group, *n* (%)	
<18	0 (0.0)	4 (2.6)	4 (2.4)	0 (0.0)
18–34	47 (47.0)	73 (48.7)	68 (41.5)	2 (22.2)
35–59 ***n***	26 (26.0)	64 (42.7)	78 (47.6)	3 (33.3)
≥60	27 (27.0)	9 (6.0)	14 (8.5)	4 (44.5)
Testing methods, *n* (%)	
Non-specific tests, (%)	
RPR card test	13 (50.0)	27 (79.4)	36 (67.9)	2 (66.7)
Agglutination method	4 (15.4)	3 (8.8)	10 (18.9)	0 (0.0)
VDRL slide test	1 (3.8)	0 (0)	1 (1.9)	0 (0.0)
Automated method	8 (30.8)	4 (11.8)	6 (11.3)	1 (33.3)
Specific tests, *n* (%)	
TP-PA	21 (84.0)	26 (92.9)	49 (89.1)	3 (100.0)
FTA-ABS	4 (16.0)	2 (7.1)	6 (10.9)	0 (0.0)

## Discussion

The nearly 10-fold increase in syphilis incidence in Nagasaki Prefecture from 2014 to 2023, with marked acceleration after 2021, mirrors the resurgence observed in other high-income countries (6) and highlights the growing public health burden of syphilis (9). The disproportionate burden among young adults, particularly those aged 18–34 years, and among males is also consistent with global disease burden patterns (10). Potential determinants of this epidemiological shift include pandemic-related behavioral changes, altered social contact patterns, and improved case detection and reporting.

The pronounced increase in syphilis cases among individuals aged 18–34 years aligns with the epidemiology of sexually transmitted infections in sexually active populations globally (4). This demographic transition can be attributed to evolving patterns of sexual behavior, particularly the proliferation of mobile social platforms and dating applications that have significantly increased the incidence of casual sexual encounters and unprotected sexual practices (11). Conversely, the declining proportion of syphilis cases among individuals aged 35–59 years suggests a potential structural demographic shift in disease burden, possibly reflecting enhanced risk awareness regarding sexually transmitted infections and improved preventive behaviors within this cohort. Emerging evidence points to a rising STI burden among older adults, likely driven by limited sexual health education and longstanding communication barriers between patients and providers, which may result in missed opportunities for prevention, diagnosis, and care (12, 13).

The gender trend analysis revealed that the male-to-female ratio of syphilis cases decreased from 2:1 in 2014 to 1.72:1 in 2023. This change may reflect increasing transmission in heterosexual populations and/or higher detection rates among women through antenatal and other healthcare services (14). Consistent with previous studies, the gender distribution of syphilis remains significantly imbalanced, with a higher proportion of male infections, particularly in the 35–59 age group, where the increase is more pronounced (15). This gender disparity reflects the continued prevalence of high-risk sexual behaviors, especially among men who have sex with men (MSM). Syphilis prevention and control efforts should therefore address both heterosexual and MSM populations through targeted interventions based on their distinct epidemiological characteristics and risk profiles. Among male cases with documented exposure history, 60.0% (81/135) reported using commercial sex services, underscoring the role of sex industry involvement in transmission. It should be noted that data on sex industry exposure were only systematically collected from 2019 onward, suggesting that the contribution of sex industry-related exposure may have been underestimated in earlier years. Additionally, six cases of HIV co-infection were identified among patients with syphilis, highlighting the important intersection between these infections. These findings emphasize the importance of targeted interventions for high-risk populations. Given the heterogeneity of sex worker populations and work environments, a more refined approach is necessary to reduce STI risk effectively (16). Preventive strategies should therefore extend beyond venue-based establishments to target harder-to-reach, non-venue-based sectors, such as street-based work, delivery-type sexual services, and individuals involved in online-facilitated sexual transactions (17). Enhanced surveillance should incorporate targeted outreach, digital health initiatives, extended testing hours, and the promotion of regular screening for individuals at increased risk and their partners.

Syphilis cases were geographically concentrated in urban areas, particularly Nagasaki City, Sasebo City, and Isahaya City. This pattern may reflect higher population density and more complex sexual contact networks in these settings. In contrast, regions with lower incidence rates, especially remote islands, may face challenges including underreporting, unequal access to healthcare, or inconsistent surveillance. Further research is needed to identify and address diagnostic and treatment barriers in these areas.

The dramatic acceleration of syphilis cases in the post-COVID-19 period (2021–2023) aligns with the gradual relaxation of social restrictions in Japan. The APC accelerated from 11.83 (2014–2021) to 99.69% (2021–2023), accompanied by a 3.4-fold increase in average annual case numbers and a significant shift toward younger age groups; the median age decreased by 5 years, and the proportion of cases aged 18–34 years increased by 12.3 percentage points (*P* = 0.017). This disproportionate impact on younger populations likely reflects resumed social mixing, nightlife activities, and expanded sexual networks following the easing of pandemic measures (18). Pandemic-related disruptions to healthcare services likely contributed to diagnostic delays and treatment gaps, amplifying subsequent case surges (19). The relaxation of social restrictions may also have prompted a rebound in sexual activity, potentially facilitated by increased use of online dating platforms. Notably, Japan experienced a substantially larger resurgence in 2022, with a 66.2% increase relative to 2020, than the more moderate increases reported in China and New Zealand (20). The sustained high proportion of primary and secondary syphilis throughout the study period underscores the continued importance of early detection and the ongoing potential for transmission.

The increase in asymptomatic syphilis cases and the re-emergence of late syphilis suggest substantial delays in diagnosis and treatment. Although asymptomatic syphilis presents no clinical manifestations, it may contribute to ongoing transmission, making routine screening among high-risk populations essential for early detection and interruption of transmission chains. Proactive screening and timely treatment are critical to prevent progression to late-stage disease and to mitigate broader public health consequences. A comprehensive response requires integrating public health education, expanded surveillance, and individualized medical interventions. Evidence from Gomes et al. (21) indicates that targeted prevention among those aged 25–34 years, individuals with lower educational attainment, and unmarried persons can significantly reduce infection rates.

This study has several limitations. First, as with all passive surveillance systems, reported cases likely underestimate true incidence because of missed diagnoses and underreporting. Second, the absence of detailed behavioral and socioeconomic data limited comprehensive risk factor assessment. Third, data on sex industry involvement were only systematically collected from 2019 onward, limiting comparisons across the full study period. Future research should explore ways to expand serological screening coverage and testing uptake among populations at increased risk (22), while improving the collection of demographic and mobility-related data to better elucidate the observed epidemiological trends.

## Conclusion

Syphilis incidence in Nagasaki Prefecture increased markedly between 2014 and 2023, with a clear acceleration after 2021 that disproportionately affected young adults and was accompanied by substantial sex industry-related exposure. These findings underscore the need for integrated, targeted public health responses, including risk-stratified screening, digital sexual health promotion, and coordinated HIV-syphilis surveillance, particularly in urban centers such as Nagasaki City.

## Data Availability

The raw data supporting the conclusions of this article will be made available by the authors, without undue reservation.
